# Identification of putative biomarkers for Infantile Hemangiomas and Propranolol treatment via data integration

**DOI:** 10.1038/s41598-020-60025-2

**Published:** 2020-02-24

**Authors:** Horacio Gomez-Acevedo, Yuemeng Dai, Graham Strub, Carrie Shawber, June K. Wu, Gresham T. Richter

**Affiliations:** 1 Department of Biomedical Informatics, University of Arkansas for Medical Sciences, Little Rock, Arkansas USA; 2 Mesquite Rehabilitation Institute, Mesquite, Texas USA; 3 Department of Otolaryngology, University of Arkansas for Medical Sciences, Little Rock, Arkansas USA; 4 Department of Surgery, New York-Presbyterian/Morgan Stanley Children’s Hospital, Columbia University, New York, New York, USA; 5 Department of Reproductive Sciences in Obstetrics & Gynecology and Surgery, Columbia University, New York, New York, USA; 6 Arkansas Children’s Hospital, Little Rock, Arkansas USA

**Keywords:** Data integration, Gene expression, Predictive markers

## Abstract

Infantile hemangiomas (IHs) are the most common benign tumors in early childhood. They show a distinctive mechanism of tumor growth in which a rapid proliferative phase is followed by a regression phase (involution). Propranolol is an approved treatment for IHs, but its mechanism of action remains unclear. We integrated and harmonized microRNA and mRNA transcriptome data from newly generated microarray data on IHs with publicly available data on toxicological transcriptomics from propranolol exposure, and with microRNA data from IHs and propranolol exposure. We identified subsets of putative biomarkers for proliferation and involution as well as a small set of putative biomarkers for propranolol’s mechanism of action for IHs, namely *EPAS1*, *LASP1*, *SLC25A23*, *MYO1B*, and *ALDH1A1*. Based on our integrative data approach and confirmatory experiments, we concluded that hypoxia in IHs is regulated by EPAS1 (HIF-2α) instead of HIF-1α, and also that propranolol-induced apoptosis in endothelial cells may occur via mitochondrial stress.

## Introduction

Infantile hemangiomas (IHs) are the most common benign tumor of the vascular endothelium in infants and are present in 4 to 10% of children younger than 12 months^1,2^. They manifest in any part of the body but around 60% of the cases arise in the head and neck^3^. IHs have a unique growth pattern that is divided into three phases: proliferation, quiescence and involution^4^. Despite the fact that the majority of IHs involute completely by 7 years of age, their hyper-proliferation can cause significant functional and disfiguring consequences and morbidities such as ulceration, bleeding, and pain. It is estimated that up to 40% of the lesions will require either surgical or medical intervention^3,5^.

Histologically, IHs in the proliferative phase show a lack of organization of blood vessels with densely packed capillaries composed of immature endothelial cells surrounded by pericytes, whereas during involution the capillaries are replaced with fatty and fibrous tissue^6^. These findings suggest that the proliferation phase is orchestrated by angiogenesis or growth factors that lead to high mitotic rates. Among such markers reported in the literature are vascular endothelial growth factor A (VEGF-A)^7^, basic fibroblast growth factor (bFGF), proliferating cell nuclear antigen (PCNA), type IV collagenase^8^, and insulin-like growth factor 2 (IGF-2)^9^. On the other hand, during the involution phase there is a high expression of antiangiogenic factors (e.g., tissue inhibitor of metalloproteinase TIMP1)^8^ and increasing apoptosis of endothelial cells^10^. However, high expression of growth factors in both the proliferating and involution phases (e.g. bFGF) makes it difficult to clearly identify causative factors for IHs’ development. From the clinical perspective, it is important to find biomarkers that robustly differentiate between proliferation and involution phases on IHs, with the hope that those biomarkers will guide medical decisions towards optimal treatments.

Propranolol is a non-selective $\beta$-adrenergic receptor antagonist originally used to treat high blood pressure that has been re-purposed for treatment of IHs and since has become the first line therapy^11^. Despite its prompt action, which is visually evident by the color change in the hemangioma, propranolol’s mechanism of action is not totally understood. Propranolol accelerates the transition of hemangiomas from proliferative to involution stages in up to 90% of cases^5^. However, several studies in IHs have reported rebound incidence or discontinuation of propranolol therapy due to side effects. More specifically, in a multi-center study of 997 IHs patients treated with propranolol Shah *et al*.^12^ found that the percentage of patients with major rebound growth was 15.7%, and rebound risk after discontinuation or tapering of propranolol were greater in female (OR = 1.67, p-value = 0.03), and in patients having deep IH morphology (OR = 3.3, p-value < 0.001). In a single center study, Shehata *et al*.^13^ observed 6% incidence of late rebound after successful treatment of IH with propranolol for an average of 10 months (n = 212). The PITCH^14^ study that consisted of 1097 children reported that 3.8% of the cohort ceased treatment due to side effects. In a case series, Pratico *et al*.^15^ reported that about 10% of patients with Infantile Cutaneous Hemangiomas treated with propranolol have re-growth, slow improvement or failure. Léaute-Labrèze *et al*.^16^ found that oral propranolol treatment with a dose up to 3 mg/kg per day for an average of 6 months is well tolerated, however treatment monitoring is necessary to eliminate well-known adverse effects (hypotension 0.2–3.1%, bradycardia 0.2–1.4%, bronchiolitis 0.3–6.2%) Thus, despite the efficacy of propranolol for IHs, it is necessary to understand the mechanisms by which propranolol induces involution in IHs to identify biomarkers of treatment efficacy to minimize its side effects and limit risk of post-treatment rebound growth. It is worth mentioning that propranolol has been recently investigated for the treatment of epithelial malignancies^17^ and breast cancer^18^. Therefore, a clear understanding of the molecular processes triggered by propranolol would expand its re-purposing value to other illnesses.

Here we present one temporal experiment that compares various IH phases with propranolol treatment at the mRNA level. To strengthen our findings, we integrate our results with other publicly available datasets that have investigated the microRNA or mRNA profile of propranolol or hemangiomas at specific stages. From our data integration, we identified gene sets that were consistently found in those experiments, and we discuss their relevance to IH development and treatment. Our integrative strategy and analysis therein challenge previous hypothesis of IHs development and propranolol mechanisms of action.

## Results

**Figure 1:**
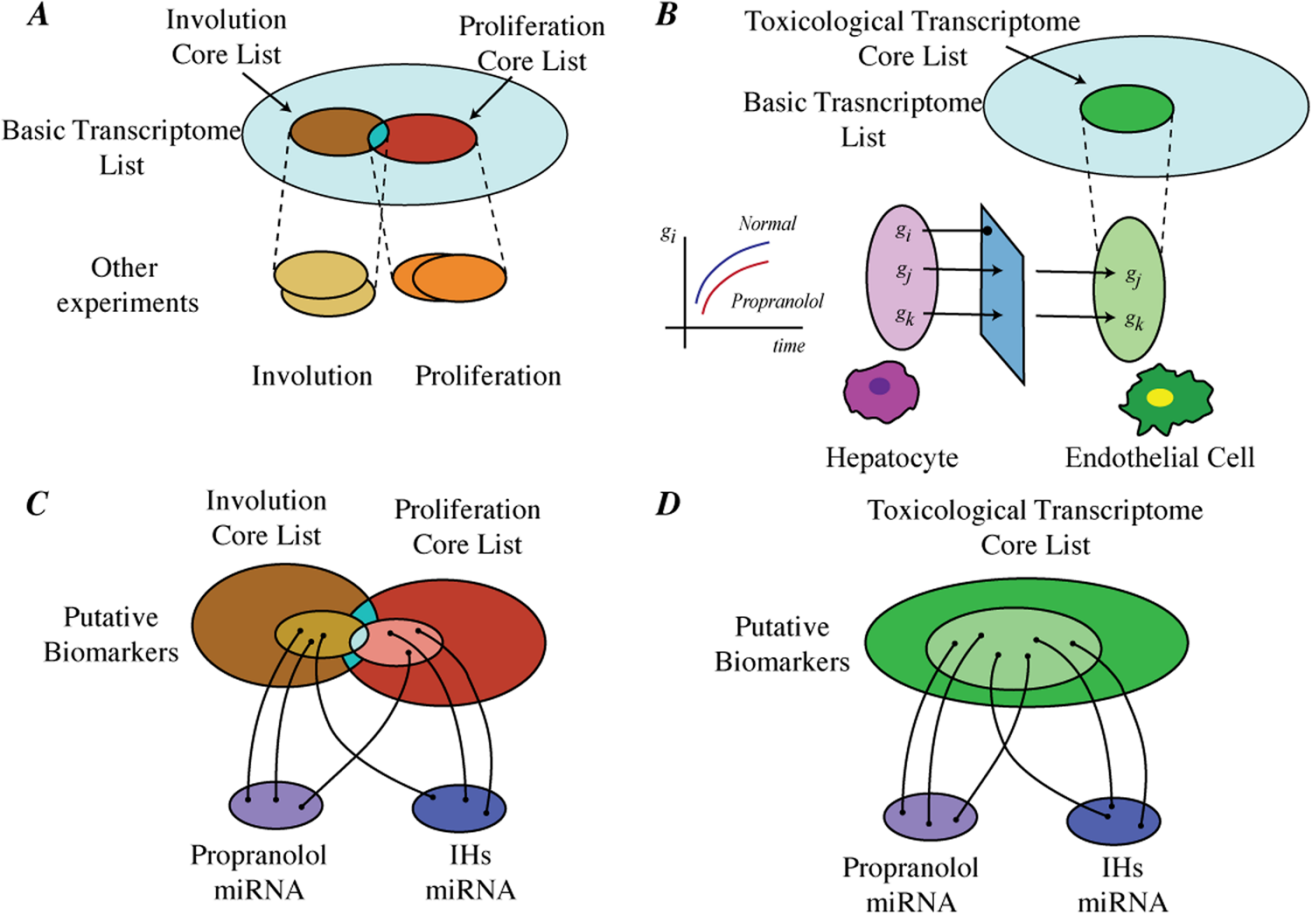
Schematic Representation of the Integrative Approach. (**A**) The basic transcriptome list was complemented with other experiments on IHs that distinguished the two phases of the disease. (**B**) Toxicology experiments on propranolol were provided differentially expressed genes that were further selected based on baseline mRNA expression using ENCODE datasets, and later intersected with the basic transcriptome list. (**C**) MicroRNA experiments from propranolol exposure or reported on IHs were mapped to the core lists and produced putative biomarkers. (**D**) Same microRNA strategy was used with the toxicological transcriptome core list to select putative biomarkers.

Our research strategy consisted of a compilation and integration of data from different sources and placed them into layers: IH transcriptome, toxicological transcriptome, and microRNA (see Fig. 1). The selection of genes from the IH transcriptome layer was based on our microarray temporal experiment of IHs and complemented by other reports of potential gene regulators of hemangioma growth and involution. The toxicological transcriptome consisted of genes whose mRNA expression significantly changed in the liver of rats exposed to propranolol^19^. To harmonize data from different tissues, we eliminated liver constitutive genes by comparing basal gene expressions hepatocytes with a group of endothelial cells selected from the ENCODE project^20^. Then, by comparing the IHs and toxicological transcriptome layers, we obtained our *basic gene set*. We mapped this basic gene set to microRNAs that have been reported to interact with such genes using the probabilistic scoring method described in TargetScore^21^. To reduce the possible number of false positive mRNA-miRNA interactions, we compared the mapped miRNA with two datasets of differentially expressed miRNA reported in the literature. One such set consisted of differentially expressed miRNA due to propranolol exposure^22^, whereas the other reported dysregulated miRNA in IHs as compared to other vascular malformations^23^. The integration of these miRNA datasets provided us with a *basic miRNA set* that is representative of the proliferation and involution phase. Our basic sets constitute putative mRNA or miRNA biomarkers for IH development that were crossed-referenced with results from experiments of propranolol treatment.

### IH’s transcriptome layer

**Figure 2:**
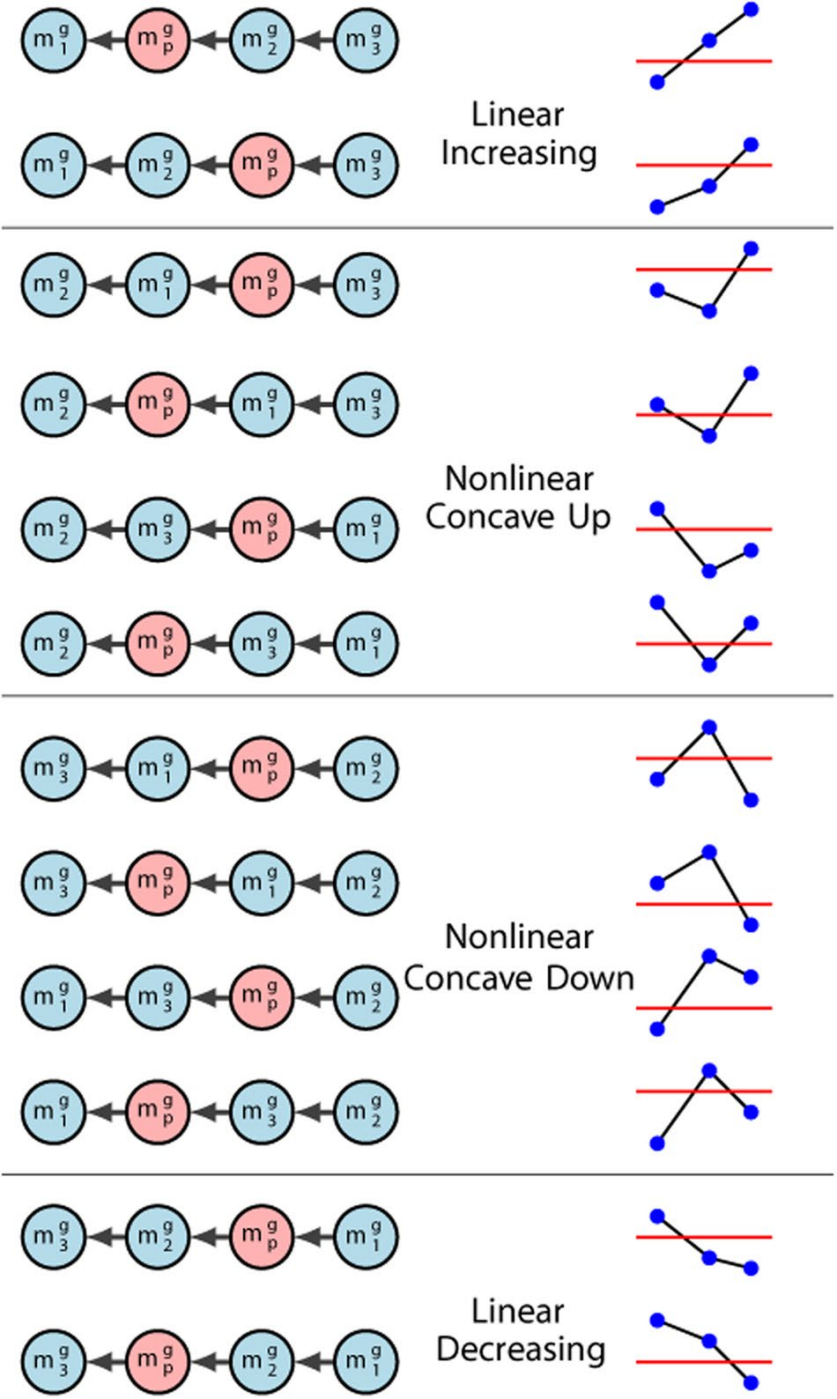
Gene expression profiles of the short time series of IHs and propranolol. On the left, the directed graphs (with direction given by the $\le$ relation) representing all combinations considered based on the mean expression levels for a gene $g$ on IHs samples at times 6 mo (${m}_{1}^{g}$), 12 mo (${m}_{2}^{g}$), and 24 mo (${m}_{3}^{i}$), and propranolol treatment ${m}_{p}^{g}$ with respect to controls. The graphical representation of the right shows propranolol mean expression (red line) with the interpolation of IHs mean expression values (blue circles).

The baseline for this layer consisted of a microarray experiment that we conducted with samples from infants with IHs at ages of 6, 12 and 24 months, plus IH samples from children treated with propranolol. The control group for microarray comparison consisted of samples taken from skin and subcutaneous tissue from healthy children. Samples from 6 month old infants were considered in proliferative stage, whereas samples at 24 months were considered in the involution stage as it has been reported clinically^4,8,24^. Our main interest was to determine genes whose mean expression levels have significantly changed during hemangioma progression from one developmental point to the next, but also those genes that changed within the range of values observed with propranolol treatment. More specifically, if we denote ${m}_{i}^{g}$ the mean expression values of the gene $g$ for hemangiomas at 6 mo, 12 mo, and 24 mo ($i=1,2,3$, respectively), and ${m}_{0}^{g}$ (resp. ${m}_{p}^{g}$) the corresponding values for control and propranolol samples respectively, then we selected probes that showed significant expression either between ${m}_{i-1}^{g}$ and ${m}_{i}^{g}$ or between ${m}_{p}^{g}$ and ${m}_{0}^{g}$ under a t-test with a FDR-adjusted $p$-value ≤ 0.05. We arranged the mean values of the selected genes in an ascending order, and the possible combinatorial arrangements are depicted on the left panel in Fig. 2. We classified the genes depending on the intersection of the propranolol level with the linear interpolation of the ${m}_{i}$ values. First, we eliminated genes whose ${m}_{p}$-levels are outside the range of the linear interpolation function, since those genes may be targeting other processes not related to the effect of propranolol on hemangiomas progression. This analysis gave us a list of 325 genes that have significantly different expression levels between hemangioma phases and with propranolol therapy. Henceforth, we will call this 325 gene list the *basic IH transcriptome list*.

We combined our findings with other mRNA studies on IHs that tried to identify genes whose expression levels changed between the proliferating and involuting phases. Calicchio *et al*.^25^ conducted a microarray (Affymetrix Human X3P GeneChip) experiment of proliferating and involuting hemangiomas from formalin-fixed samples using laser capture microdissection to select only the lesioned vessels. Placenta samples were selected as controls since the vasculature of placenta has been hypothesized to come from a common cell progenitor. In their analysis, 843 probes were differentially expressed in the proliferating phase with respect to controls at $p$-value ≤ 0.05 level of significance, whereas 958 probes were found in the involution phase with the same cutoffs. After eliminating probes that either were repeated or did not have Entrez, HGNC or Ensembl annotations, we found 544 differentially expressed genes for the proliferating phase, and 573 for the involution phase. Ritter *et al*.^9^ conducted a microarray (Affymetrix U95Av2) experiment that consisted of resected hemangioma specimens that were clinically classified as being in proliferation, plateau or involuting phases. They reported 112 probes that had 3-fold increase (or decrease) of expression between the proliferating and involuting phases, but no statistical test was reported for probe selection. After elimination of repeated probes or ones without proper annotation there were 101 genes considered from that dataset. We further classified a gene as proliferating if the mean expression level was higher in proliferating than in involuting samples; conversely, we classified a gene as involuting if the mean expression level was highter in involuting samples than proliferative ones. We pasted together the proliferating phase from Rittier *et al*. and Calicchio *et al*., and we intersected those genes with our basic IH transcriptome list. Henceforth, the results from these intersections will be referred to as *proliferating*-*IH transcriptome core list* (Table 1). Similarly, we intersected the involuting list of genes from the previous reports with our basic IHs transcriptome list to obtain the *involuting-IH transcriptome core list* (Table 2).

**Table 1:** Proliferating Transcriptome Core List. Codes for response are: (LD) linear decreasing, (LI) linear increasing, (CD) nonlinear concave down, and (CU) nonlinear concave up.

HGNC SYMBOL	RESPONSE	ENSEMBL ID	ENTREZ	DEFINITION
ALCAM	CU	ENSG00000170017	214	Activated leukocyte cell adhesion molecule.
ANGPT2	LD	ENSG00000091879	285	Angiopoietin 2.
ATP1B1	LU	ENSG00000143153	481	ATPase, Na+/K+ transporting subunit beta 1.
CCND1	CD	ENSG00000110092	595	Cyclin D1.
BST2	CD	ENSG00000130303	684	Bone marrow stromal cell antigen 2.
SERPINH1	LD	ENSG00000149257	871	Serpin family H member 1.
CDH5	CD	ENSG00000179776	1003	Cadherin 5.
COL4A1	CD	ENSG00000187498	1282	Collagen, type IV, alpha 1.
COL4A2	LD	ENSG00000134871	1284	Collagen, type IV, alpha 2.
FMOD	CU	ENSG00000122176	2331	Fibromodulin.
GATM	CU	ENSG00000171766	2628	Glycine amidinotransferase.
GPER1	CD	ENSG00000164850	2852	G protein-coupled estrogen receptor 1.
HLX	CD	ENSG00000136630	3142	H2.0-like homeobox.
ITGA1	LD	ENSG00000213949	3672	Integrin, alpha 1.
LGALS1	LD	ENSG00000100097	3956	Lectin, galactoside-binding, soluble, 1.
NOTCH4	CD	ENSG00000204301	4855	Notch homolog 4.
OAZ2	LD	ENSG00000180304	4947	Ornithine decarboxylase antizyme 2.
PDGFRB	LD	ENSG00000113721	5159	Platelet-derived growth factor receptor beta.
PECAM1	CD	ENSG00000261371	5175	Platelet/endothelial cell adhesion molecule.
PPL	CU	ENSG00000118898	5493	Periplakin.
THY1	CD	ENSG00000154096	7070	Thy-1 cell surface antigen.
PXDN	LD	ENSG00000130508	7837	Peroxidasin homolog.
APLN	CD	ENSG00000171388	8862	Apelin.
PPM1F	CD	ENSG00000100034	9647	Protein phosphatase 1F.
SH2D3C	CD	ENSG00000095370	10044	SH2 domain containing 3C.
SPINT2	CU	ENSG00000167642	10653	Serine peptidase inhibitor, Kunitz type, 2.
VASH1	LD	ENSG00000071246	22846	Vasohibin 1.
ABLIM3	CD	ENSG00000173210	22885	Actin binding LIM protein family, member 3.
RBFOX2	LD	ENSG00000277564	23543	RNA binding motif protein 9.
MXRA5	LD	ENSG00000101825	25878	Matrix-remodelling associated 5.
MYLIP	CD	ENSG00000007944	29116	Myosin regulatory light chain interacting protein.
RASL12	CD	ENSG00000103710	51285	RAS-like, family 12.
JAM3	CD	ENSG00000166086	83700	Junctional adhesion molecule 3.
LINGO1	CD	ENSG00000169783	84894	Leucine rich repeat and Ig domain containing 1.
ZBTB46	CD	ENSG00000130584	140685	Zinc finger and BTB domain containing 46.

**Table 2:** Involution Transcriptome Core List. Codes for response are: (LD) linear decreasing, (LI) linear increasing, (CD) nonlinear concave down, and (CU) nonlinear concave up.

HGNC SYMBOL	RESPONSE	ENSEMBL ID	ENTREZ	DEFINITION
ANG	CU	ENSG00000214274	283	Angiogenin, ribonuclease, RNase A family, 5.
ATP1B1	LI	ENSG00000143153	481	ATPase, Na+/K+ transporting, beta 1 polypeptide.
BCL6	CU	ENSG00000113916	604	B-cell CLL/lymphoma 6 (zinc finger protein 51).
CD9	LI	ENSG00000010278	928	CD9 molecule.
DEFB1	CU	ENSG00000164825	1672	Defensin, beta 1.
FBLN1	LI	ENSG00000077942	2192	Fibulin 1.
GAS1	CU	ENSG00000180447	2619	Growth arrest-specific 1.
IGFBP7	CD	ENSG00000163453	3490	Insulin-like growth factor binding protein 7.
PPL	CU	ENSG00000118898	5493	Periplakin.
RBMS1	CD	ENSG00000153250	5937	RNA binding motif, single stranded interacting protein 1.
SFRP1	LI	ENSG00000104332	6422	Secreted frizzled-related protein 1.
ASAP2	CD	ENSG00000151693	8853	Development and differentiation enhancing factor 2.
LDB2	CD	ENSG00000169744	9079	LIM domain binding 2.
NDRG1	LI	ENSG00000104419	10397	N-myc downstream regulated gene 1.

The drastic down-regulation of the Serpin family E member 2 (*SERPINE2*) in both the proliferative and involution phases of hemangiomas with respect to placenta tissue reported by Calicchio^25^ was probably an artifact and not necessarily related to hemangioma development since RNA-seq experiments from the Human Protein Atlas^26^ show very high expression levels (TMP values) in placental tissue under homeostatic conditions. Therefore, we eliminated that gene from both Tables 1 and 2.

### Toxicological transcriptome layer

From the project “Transcriptional profiling of rat liver after short-term administration of carcinogenic and non-carcinogenic chemicals” (GSE68110), we downloaded time series data on rat livers treated with propranolol for up to 14 days or controls, and reanalyzed the molecular profiling, which was obtained with Affymetrix RG-230A microarrays. We assumed a smooth expression change over time for propranolol or vehicle treatments, thus we fit the temporal trend using regression splines with 3 knots with the *splines* package in R. We then fit separate curves for the propranolol and control groups using *limma* package. Three hundred and one genes showed statistically significant differences in their time trends between propranolol and control groups under a moderate $F$ test with 3 degrees of freedom and a FDR-ajusted $p$-value ≤ 0.05.

**Figure 3:**
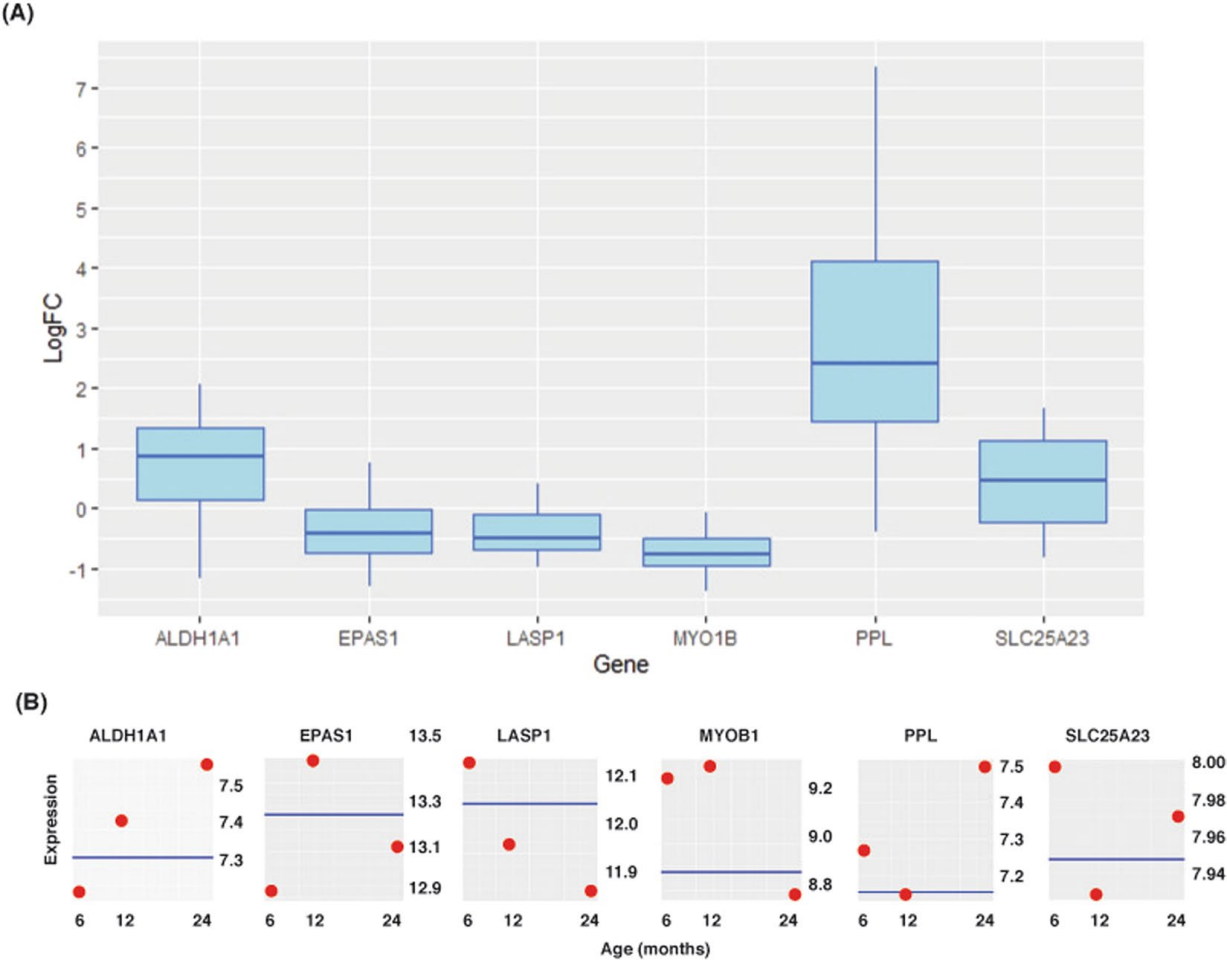
Relative gene expression changes by RT-PCR. An independent set of IH samples in the involution phase (more than 24 mo) and in the proliferation phase (less than 6mo) with n = 6 per group. (**A**) Logarithmic fold change of gene expression between IHs in the involution phase (numerator) and IHs in the proliferation phase (denominator). (**B**) Average gene expression from the microarray experiment (blue line represents the mean level of expression in the propranolol group).

To harmonize the previous transcriptome analysis from animals into humans using propranolol for IH treatment, we first matched rat annotation into human annotation using Ensembl symbols through the *biomaRt* package of Bioconductor, and dropped probes that have no human gene homologs. Secondly, we eliminated probes whose expression levels in hepatocytes are too high (or low) in comparison to homeostatic expression of endothelial cells, as those probes may induce artifacts that are not directly affected by propranolol but may be due to their *constitutive expression*. More specifically, we downloaded RNA-seq data from the ENCODE project^20^ from hepatocytes and different primary endothelial cells (see Table 3). From those datasets, we selected genes whose mean raw-count values between hepatocytes and endothelial cells had a $|{\log }_{2}$(fold change)| lower than 2. From the 301 previously found genes, only 230 had proper annotation and comparable dynamic range of raw expression between hepatocytes and endothelial cells. The resulting list of genes was then intersected with the basic IH transcriptome list, yielding to only 5 genes: Aldehyde dehydrogenase 1 family member A1 (*ALDH1A1*), Endothelial PAS domain protein 1 (*EPAS1* also known as *HIF-2*$\alpha$), LIM and SH3 protein 1 (*LASP1*), Myosin IB (*MYO1B*), and the Solute carrier family 25 member 23 (*SLC25A23*), and henceforth we will refer to this set of genes as *toxicological transcriptome core list* (see Table 4). We confirmed that relative gene expression changes during proliferation and involution by RT-PCR (see Fig. 3)

**Table 3:** ENCODE RNAseq experiments used for basic expression comparison. (*) Primary cell.

Cell Type	ENCODE accession file
Hepatocyte	ENCFF072KSA
Hepatocyte	ENCFF491FPY
Dermis blood vessel endothelial*	ENCFF620THF
Dermis blood vessel endothelial*	ENCFF699QEJ
Dermis lymphatic vessel endothelial*	ENCFF985PDI
Dermis lymphatic vessel endothelial*	ENCFF797YZO
Dermis microvascular lymphatic vessel endothelial*	ENCFF764AOQ
Dermis microvascular lymphatic vessel endothelial*	ENCFF110UGQ
Vein endothelial*	ENCFF592KDP
Vein endothelial*	ENCFF361WEZ

**Table 4:** Toxicological Transcriptome Core List. Codes for response are: (LD) linear decreasing, (LI) linear increasing, (CD) nonlinear concave down, and (CU) nonlinear concave up.

HGNC SYMBOL	RESPONSE	ENSEMBL ID	ENTREZ	DEFINITION
ALDH1A1	LI	ENSG00000165092	216	Aldehyde dehydrogenase 1 family member A1
EPAS1	CU	ENSG00000116016	2034	Endothelial PAS domain protein 1
LASP1	LD	ENSG00000002834	3927	LIM and SH3 protein 1
SLC25A23	CD	ENSG00000125648	79085	Solute carrier family 25 member 23
MYO1B	CU	ENSG00000128641	4430	Myosin IB

### MicroRNA layer

We re-analyzed previously reported microRNA data from CD-1 mice exposed to propranolol for up to 14 days^27^ (GSE67943). Since the data was obtained with Agilent mouse miRNA microarray, we used the bioconductor *AgiMicroRna* package to extract, process and normalize the data followed by an analysis with the *limma* package to compare controls to propranolol-exposed mice regardless of gender. A list of 9 microRNAs were differentially expressed at a FDR-adjusted $p$-value < 0.05 (see Table 5).

**Table 5:** MicroRNA found to be affected by propranolol exposure. *Two mature forms 5p and 3p.

Mouse miRNA	Human miRNA
mmu-miR-697	hsa-miR-517a-3p
mmu-miR-212*	hsa-miR-517c-3p
mmu-miR-202-3p	hsa-miR-518d-5p
mmu-miR-872*	hsa-miR-519a-3p
mmu-miR-1904	hsa-miR-520c-3p
mmu-miR-689^†^	hsa-miR-525-5p
mmu-miR-1897-5p	hsa-miR-126-3p
mmu-miR-705	hsa-miR-126-5p
mmu-miR-1892	

^†^Dead entry on miRbase database, and thus not considered for analysis.

Reports on the microRNA effects of propranolol on IHs are scarce and two reports on IHs were included in this study. From Strub *et al*.^23^, we selected members of the C19MC microRNAs found in blood that showed changes between pre-treatment and 1 or 6 months of propranolol exposure. Biswas *et al*.^28^ reported that microRNA 126 as a biomarker for the proliferative phase of infantile hemangiomas; however, it was not clear from their report which mature form (miR-126-3p or miR-126-5p) was used for their comparisons. In both of the previous studies, microRNA quantification was performed with RT-PCR that precluded us from re-analyzing raw data for better data integration. Thus, we combined the microRNAs mentioned in those papers and queried *miRbase* database^29^ to verify microRNA annotation (see Table 5). The combined list of microRNAs is referred to as *microRNA core list*.

**Figure 4:**
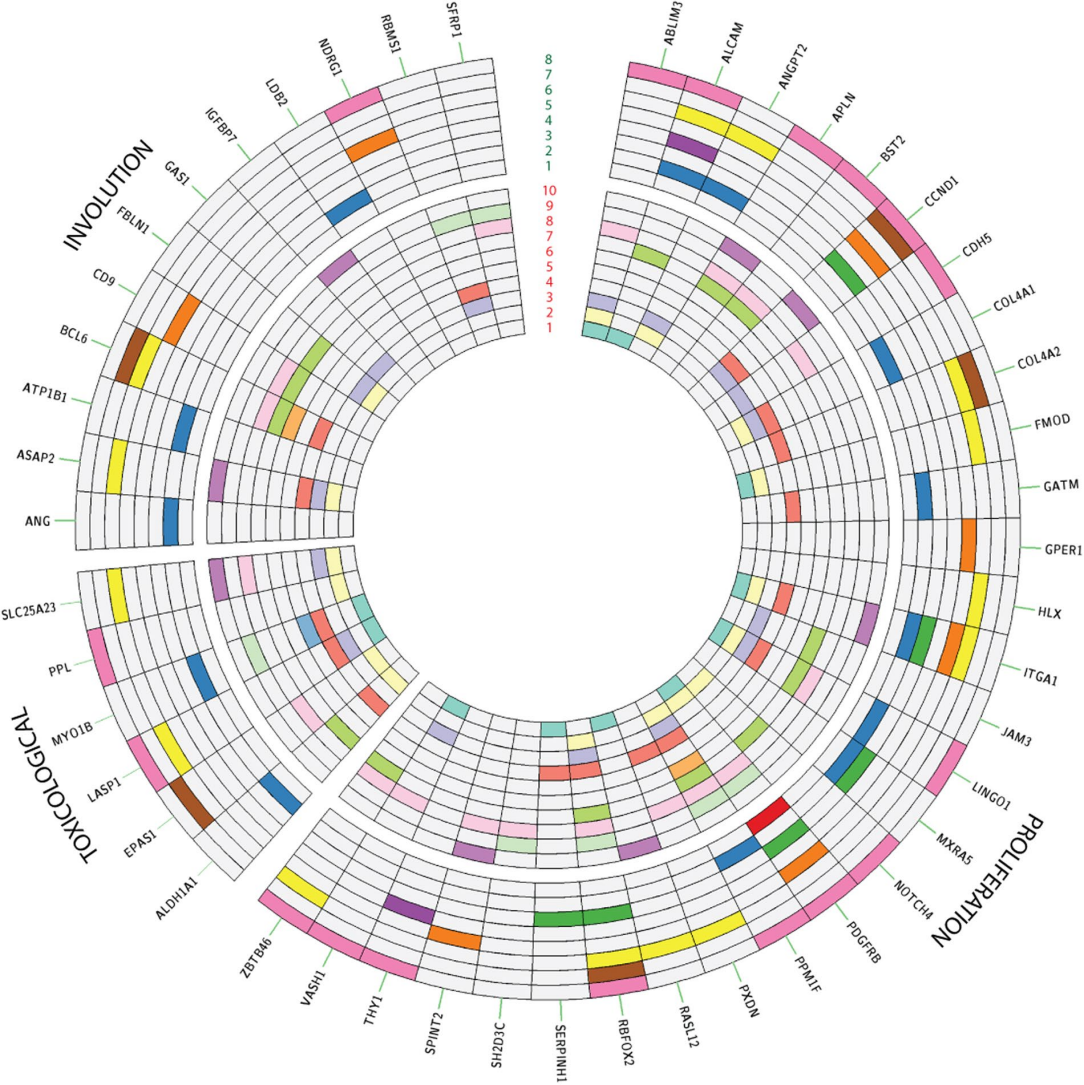
Putative Biomarkers. Each wedge contains the corresponding genes that were mapped to their corresponding microRNA. The inner circles correspond to murine mmu-miR (1) 875-5p, (2) 872-3p, (3) 705, (4) 697, (5) 212-5p, (6) 212-3p, (7) 202-2p, (8) 1887-5p, (9) 1892, and (10) 1094, whereas the outer correspond to human hsa-miR: (1) 525-5p, (2) 520c-3p, (3) 519a-3p, (4) 518d-5p, (5) 517c-3p, (6) 517a-3p, (7) 126-5p, and (8) 126-3p. Periplakin (PPL) was the only common biomarker on the involution and proliferation list.

From the microRNA core list, we used a *backwards* mapping (miRNA$\to$ mRNA) as an alternative method to corroborate the role of specific genes found in our previous core lists. More specifically, we mapped each microRNA to putative target genes using *TargetScan* v 7.2.^30^ for human and mouse. We further selected only targets that have matching human-mouse homologs. As we observe in Fig. 4, all the toxicological transcriptome core was mapped by at least three miRNAs, whereas 86% of the proliferation-phase and 93% of the involution-phase genes were mapped by at least one miRNAs. Henceforth, we will refer to genes found in the core lists that were mapped by microRNAs as **putative biomarkers**.

Our integrative analysis showed that hsa-miR-518d-tp, hsa-miR-519a-3p, and hsa-miR-525-5p are solely targeting putative IH transcriptome proliferation biomarkers, namely *PDGFB*, *CCND1*, *ALCAM*, *MXRA5*, and *ITGA1* (see Fig. 4). On the other hand, only mmu-miR-212-5p uniquely targets the toxicological transcriptome putative biomarker *MYO1B*, and none of the microRNAs investigated mapped uniquely to any of the putative involution genomics biomarkers. Thus, it is more likely that a robust signature based on microRNAs would include a combination of microRNAs rather than a single one. For instance, Chang and colleagues^31^ investigated recurrence of IHs after propranolol treatment and predicted that hs-miR-122-3p targets only *ANGPT2*, whereas hsa-miR-758-3p targets both *ANGPT2* and *COL4A1*.

## Discussion

### Effects of propranolol on IHs

**Figure 5:**
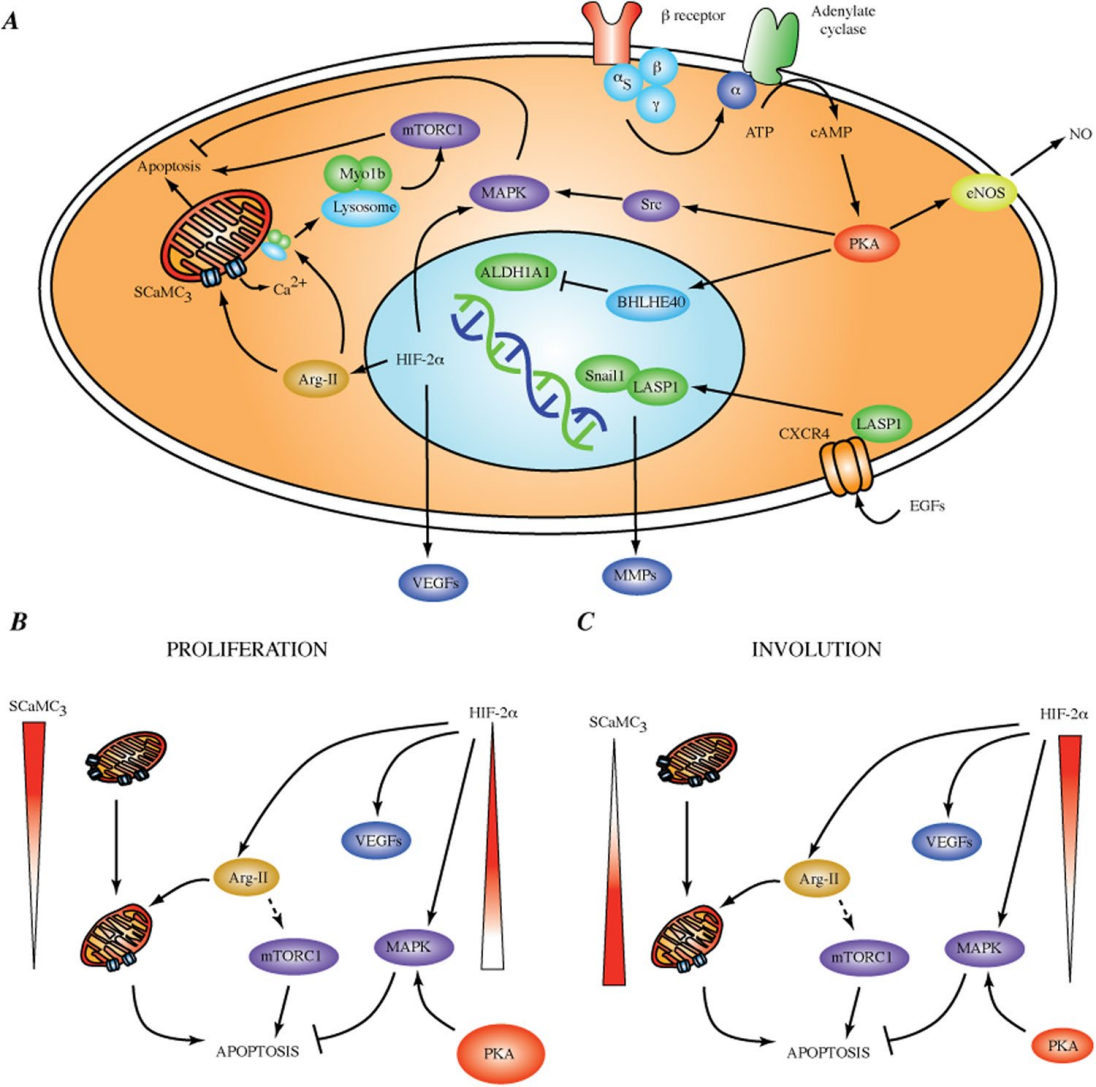
Putative Toxicological Transcriptome Biomarkers and their role on IHs and possible mechanism of action of propranolol. (**A**) Pathways affected by biomarkers. (**B**) Proliferation. High levels of PKA and SCaM${C}_{3}$ provide an antiapoptotic environment. Decline in the calcium carriers induces mitochondria stress; the up-regulation of HIF-2$\alpha$ stimulates VEGFs but it has the side effect of increasing levels of Arg-II exacerbating apoptotic pathways. (**C**) Involution. Despite the autoregulatory increase of SCaM${C}_{3}$, and ongoing production of VEGFs by HIF-2$\alpha$, the antiapoptotic signals cannot be overcome due to the lack of PKA-induced MAPK survival signals.

We proceeded to address potential propranolol mechanisms of action using our toxicological transcriptome putative biomarkers as guide (see Fig. 5 and Table 4).

### IH-propranolol and vasoconstriction

It is widely accepted that propranolol affects vasoconstriction through its antagonistic role on $\beta$-adrenergic receptors. These receptors belong to the superfamily of G-protein coupled receptors expressed by endothelial cells. Upon binding with $\beta$-adrenergic agonist, the activation of adenylyl cyclase takes place via ${G}_{S}$-proteins and results in a conversion of adenosine triphosphate into cyclic AMP (cAMP), which acts as a second messenger in the cytosol and activates the protein kinase A (PKA). PKA can in turn phosphorylate different intracellular proteins. In endothelial cells, PKA induces phosphorylation at Ser1179 on the endothelial nitric oxide synthase (eNOS), and the nitric oxide (NO) produced by eNOS diffuses to nearby vascular smooth cells causing a regulation of local blood flow due to the increased levels of cyclic guanosine monophosphate (cGMP) by soluble NO. The activation of protein kinase G (PKG) by cGMP will in turn induce vasodilation and relaxation of the vascular smooth muscle^32,33^. Thus, propranolol is expected to reduce the NO levels in the extracellular space leading to vasoconstriction, and intracellular reduction of PKA levels.

### IH-propranolol and matrix metalloproteinases (MMPs)

Matrix metalloproteinases are enzymes capable of degenerating extracellular matrices (ECM) such as collagen, fibronectin and elastin, which ultimately facilitates cell movement and migration. Recent studies have shown that over-expression of LIM and SH3 protein 1 (*LASP1*) promotes MMP activity and matrix degradation^34^. There is evidence that either knockdown or stable silencing *LASP1* reduced MMP1, 3 and 9 expression^35^. Moreover, *LASP1* can disrupt intercellular adherens junctions such as E-cadherin through the phosphorylation of LASP1 on serine 146 induced by PKA that facilitates its binding to the chemokine receptor CXCR4. Upon stimulation with chemokines (i.e., CXC12, EGF), LASP1 translocates into the nucleus and stabilizes Snail1, which ultimately will reduce the expression of E-cadherin and other junction proteins^36,37^. Thus, the downstream effect of PKA reduction by propranolol is likely to affect *LASP1*’s ability to produce MMPs, thereby hampering cell migration. We confirmed downregulation of *LASP1* during the involution phase by RT-PCR (see Fig. 3). Our analysis suggests that propranolol does not affect anti MMPs factors (i.e., TIMPs) directly but by disrupting *LASP1*.

### IH-propranolol, hypoxia and angiogenesis

It has been hypothesized that hypoxia is directly involved in the pathogenesis of infantile hemangiomas^38–40^, and clinically up to 50% exhibit local ischemia in the site where IH ultimately develop^41^. *EPAS1* mRNA stabilizes and accumulates during prolonged hypoxic conditions, and this gene encodes for the transcription factor HIF-2$\alpha$ which shares 48% of sequence identity with the master regulator of hypoxia HIF-1$\alpha$. HIF-2$\alpha$ and HIF-1$\alpha$ may play redundant roles during development as they co-localize with some common transcripts; however, they can promote the expression of distinct genes in endothelial cells^42^. HIF-2$\alpha$ levels increase after the original levels of HIF-1$\alpha$ decline in response to prolonged hypoxia in the endothelium^43^. The action of HIF-2$\alpha$ is limited to some specific cell lineages including endothelial cells, and influences angiogenesis through upregulation of VEGFA, and other common targets include the glucose transporter 1 (*GLUT1*) and erythropoietin (*EPO*)^43^. Other reports have pointed out to the role of HIF-1$\alpha$ in propranolol treatment of IHs^44^; however, our compilation of high-throughput experiments did not show significant changes in *HIF-1*$\alpha$ but repeatedly showed changes in *HIF-2*$\alpha$. We further verified that HIF-2α is upregulated during the proliferative phase and later decreased. Thus, we conclude that hypoxia in IHs is not guided by HIF-1a (as widely suggested in the literature) but by HIF-2α. In hemangioma stem cells, it was convincingly demonstrated that propranolol reduces cAMP and concomitantly MAPK signaling^45^. Thus, it may be a combination of a locally hypoxic environment and reduced cAMP levels which ultimately leads to a reduction in the production of angiogenic factors (e.g., VEGFs).

Another piece of evidence that supports the important role of hypoxia in IHs comes from the detoxifying enzyme aldehyde dehydrogenase 1 (ALDH1) that has been commonly used as a marker of stemness in breast cancer cells (BCSC), and under hypoxic conditions produces a phenotypical shift of BCSCs into a more proliferative subtype^46^. In an avascular system (i.e., cornea) it has been suggested that severe hypoxic conditions regulation of mRNA expression of *ALDH1A1* occurs via the cAMP activation of the basic helix-loop-helix family member E40 (*BHLHE40* or *STRA13*) which inhibits the E-box element on the promoter region of *ALDH1A1*^47^. This mechanism may explain the strong repression of *ALDH1A1* on early stages of hemangiomas that is later lessened as hypoxia and cAMP levels decrease. Of note, it has been reported that HIF-2$\alpha$ does not bind to the *ALDH1A1* promoter in BCSCs under hypoxic conditions^46^; however, alternative binding mechanisms may be available to differentiated endothelial cells. We confirmed that in the proliferation phase of IHs, *ALDH1A1* mRNA expression is suppressed. Taken together, these results strongly suggest that there is a locally hypoxic micro-environment in early stages of IHs development (see Fig. 3).

### IH-propranolol and cell death

Mechanistic evidence of propranolol-induced apoptosis has been reported for infantile hemangiomas. Li *et al*.^48^ found that propranolol promoted cell apoptosis via the AKT/mTOR pathway on hemangioma-derived endothelial cells isolated from proliferating IH tissues (XPTS-1). In pulmonary endothelium, the stability of HIF-2$\alpha$ increases arginase expression^49^, which in turn causes mitochondrial dysfunction through changes in membrane potential via ${{\rm{Ca}}}^{2+}$ uptake^50^. Myosin-1b (encoded by *MYO1B*) has been shown to mediate the effect of Arg-II on mTORC1 by inducing lysosome re-distribution to the cell periphery and activation of the mTORC1-S6K1 signaling that leads to mitochondrial dysfunction and apoptosis^51^. We verified that *MYO1B* is upregulated during proliferation suggesting an anti-apoptotic mechanism via Arg-II-mTORC1 axis that limits mitochondrial dysfunction.

Another important cue to clarify the role of propranolol in cell death regulation comes from the small calcium-binding mitochondrial carriers (SCaMCs), that have been shown to play an important role in cell death under different physiological conditions^52^. SCaMC-3 (encoded by the solute carriers 25A23 *SLC25A23* gene) transport adenine nucleotides in response to ${{\rm{Ca}}}^{2+}$. Under schemic conditions, it has been reported that over-expression of SCaMC appears to be a survival mechanism used by cancer cells to avoid cell death triggered by oxidative stress and ${{\rm{Ca}}}^{2+}$ excess^53,54^. Further, knocking down *SLC25A23* in HeLa cells reduces the mitochondrial ROS and cell death^55^. We observed that the relative expression of *SLC25A23* was slightly higher in the involution phase than in its proliferative counterpart (see Fig. 3). Taken together, the combination of locally hypoxic conditions during the early development of hemangiomas with high expression of SCaMC-3 may protect the cell from oxidative stress, while the lower levels of Myosin-1b would limit its impact on lysosome relocation and apoptosis via the Arg-II-mTORC1 axis and mitochondrial stress. Therefore, our data and analysis support the hypothesis that apoptosis during IH progression from proliferation to involution is related to mitochondrial responses to changes in ${{\rm{Ca}}}^{2+}$ influxes and buffering.

### Putative biomarkers and progression from proliferation to involution

The unique behavior of expansion and shrinkage occurring naturally in IHs leads to the search for two types of biomarkers: a) those biomarkers that are continuously decreasing or increasing as hemangiomas progress (and we refer to those as *linearly responsive biomarkers*), or b) those biomarkers that mimic the increase and decrease pattern of the hemangioma (we refer to those as *non-linear responsive biomarkers*). Linearly responsive biomarkers can detect unequivocally the stage of the IH (either proliferating or involuting) over a period of time, and can serve as a surrogate endpoint for therapy efficacy. On the other hand, non-linear responsive biomarkers may more closely follow the natural history of a hemangioma over time, and it is plausible that some of these biomarkers are directly affected by (or part of) the yet to be determined mechanisms driving hemangiomas.

### Linearly responsive putative proliferation biomarkers

Among the linearly responsive biomarkers for proliferation we found that all 9 of them exhibit a decreasing behavior. More specifically, these include angiopoietin 2 (*ANGPT2*), serpin family H member 1 (*SERPINH1*), integrin subunit $\alpha$ 1 (*ITGA1*), platelet derived growth factor $\beta$ receptor (*PDGFRB*), RNA binding FOX-1 homolog 2 (*RBFOX2*), matrix remodeling associated 5 (*MXRA5*), vasohibin 1 (*VASH1*), peroxidasin (*PXDN*), and collagen type IV $\alpha$ 2 chain (*COL4A2*). We proceed to briefly describe how these genes, whose expression attenuates with time, may shed light on natural history of IHs and could provide alternative measurable markers to monitor the disease progression.

#### ANGPT2

Angiopoietin 2 (Ang-2) is secreted from Weibel-Palade bodies in the vascular endothelium. Ang-2 over-expression compromises endothelial junction integrity, promotes the removal of pericytes, and prepares the vasculature for angiogenic sprouting. Lower levels of Ang-2 mRNA expression are observed in quiescent resting vasculature, although its expression can be stimulated by hypoxia^56,57^. It is important to note that Ang-2 facilitates angiogenesis only in the presence of VEGF, and absence of the latter induces endothelial cell apoptosis^58^. The pivotal role of the Ang-Tie system in controlling anti-angiogenesis, vascular remodeling, and serving as an endothelial cell switch between quiescence and growth^59^, makes Ang-2 a primary target for understanding the development of IHs.

#### COL4A2

Increased secretion and accumulation of extracellular collagen type IV in endothelial cells are important steps for angiogenesis both *in vivo* and *in vitro*^60^. Collagen IV protein network is the main constituent of the basement membrane which interacts with receptors such as integrins to control cell adhesion, proliferation and migration^61^. Mutations in *COL4A1* and *COL4A2* have been related to aneurysms^62^. The continuous decrease in gene expression of *COL4A2* may indicate reduced migration and reduced angiogenesis as expected during the involution phase. Park *et al*.^63^ have already reported changes in the extracellular matrix (ECM) composition on proliferation and involution, but unfortunately they did not investigate collagen IV. Other reports have detected collagen IV on the basement membrane by immunostaining on all stages of hemangiomas^64^.

#### ITGA1

Integrins are cell adhesion receptors that regulate cell-to-cell and cell-to-EMC interactions, thereby mediating cell movement, attachment, and other important processes. *ITGA1* encodes for the integrin $\alpha 1\beta 1$, which is widely expressed on cells that are in contact with basement membranes (e.g., pericytes and smooth muscle cells) as well as connective tissue cells (e.g., fibroblasts). Several collagens are ligands for$\,\alpha 1\beta 1$; however, integrins prefer to interact with collagen IV rather than collagen I^65^. $\alpha 1\beta 1$ receptor is able to activate the Ras/Shc/MAPK pathway promoting cell proliferation^66^. Thus, it may be possible that the combination of higher levels of collagen IV and integrin $\alpha 1\beta 1$ stimulate cell proliferation in the early stages of hemangiomas.

#### MXRA5

The adhesion proteoglycan MXRA5 (also known as adlican) has VEGF receptor activity, and has been included in a set of genes involved in extra cellular matrix remodeling^67^. Adlican appears to decrease cell adhesion in tumor fibroblasts from esophageal squamous cell carcinoma^68^. In the trophoblast cell line HTR-8/SVneo, *MXRA5* attenuated apoptosis induced by TNF-α^69^. Thus, the high expression of *MXRA5* during proliferation may remodel extracellular matrix to promote survival and proliferation. In a study of Chinese patients with non-small cell lung carcinoma^70^, frequent somatic mutations in *MXRA5* that modified the extracellular matrix remodeling were found. Using a proteomics approach, Glavey *et al*.^71^ confirmed that MXRA5 is part of the bone marrow extracellular matrix glycoproteins in normal and relapsed multiple myeloma patients, but until now changes in the endothelium by *MXRA5* expression and concomitant effects in extracellular matrix microenvironment are unknown.

#### PDGFRB

Experiments on endothelial progenitor cells (EPCs) show that PDGFR-$\beta$ plays a relevant role in proliferation and migration via p-ERK signaling pathway^72^. Moreover, it has been demonstrated that a decrease in phosphorylation of PDGFR-$\beta$ on EPCs leads to limited activation of phosphoinositide 3-kinase (PI3K)/Akt pathway, thereby reducing proliferation^73^. Physiologically, PDGFR- β also promotes proliferation of pericytes and smooth muscle cells during their recruitment to the endothelium. Of note, itraconzole (which is a common anti-fungal drug) has recently been tested as a therapy for IHs, and this drug targets PDGFR-$\beta$ activation, in turn inhibiting PI3K and Atk^74^.

#### PXDN

Peroxidasin is a member of the family of heme peroxidases. Bhave and colleagues^75^ showed that PXDN catalyzes formation of the sulfillmine ($S=N$) bond through NC1 domain of collagen IV, which in turn cross links collagen IV through its NC1 domain to form a sheet-like network for basement membranes. It is interesting that PXDN collagen IV synthesis continues even under extremely hypoxic conditions^76^. Apart from its role in the ECM network, over-expression of PXDN (also known as vascular peroxidase 1 or VSO1) during development of hypertension leads to endothelial dysfunction and attenuates NO production^77,78^.

#### RBFOX2

Rbfox2 is a member of the family of splice factors Rbfox, and the only one in the family to be expressed in the arterial endothelium^79^. Rbfox2 mediates pre-mRNA alternative splicing in vascular Cav1.2 channels. The activation of the Cav1.2 is a response to changes in intramural pressure due to blood flow changes. In an animal model, Zhou and colleagues^80^ found that knocking down Rbfox2 up-regulates Cav1.2 channel activity and leads to vasoconstriction. Further, Murphy *et al*.^81^ found that Rbfox2 is an important regulator of endothelial cells exposed to an acute reduction in blood flow. Thus, *RBFOX2* expression level serves as a physiologically-driven biomarker that in conjunction with Doppler ultrasound^28^ could trace the development of IHs based on patterns of flow velocities.

#### SERPINH1

This gene encodes the heat inducible protein HSP47, which is a member of the serpine family of proteinase inhibitors. HSP47 is crucial during pro-collagen biosynthesis and plays and important role in the secretion of decorin, fibromodulin and lumican for the assembly of the extracellular matrix^82^. In primary grade IV gioblastoma multiforme (GBM) primary cells, an over-expression of HSP47 promoted the expression of extracellular matrix (ECM)-related genes such as *COL4A2* and *MMP9*^83^. In fact, a reduced expression of MMP9 mRNA was reported in younger children (less than 1 year old) and older than 1 year after propranolol treatment^84^, however *MMP9* did not show up in our integrative approach. Thus, it may be possible that the observed reduction in MMP9 mRNA is related to decrease of HSP47 rather than a direct effect of propranolol on *MMP9*.

#### VASH1

Vasohibins are considered to be angiogenic inhibitors in endothelial cells, but interestingly vasohibin-1 exhibits a negative feedback regulation with VEGF under hypoxic or inflammatory conditions^85^. The anti-angiogenic role of VASH1 expression on endothelial cells varies depending on its location on the vascular architecture^86^. Thus, the high gene expression levels of *VASH1* during the early onset of IHs pose a paradigm for the angiogenic events. An alternative explanation for the high levels of gene expression of *VASH1* may lie in the tubulin detyrosinating properties of the vasohibins^87^. More specifically, the up-regulation of vasohibins induces the accumulation of detyrosinated $\alpha$-tubulin (Glu-tubulin), which is a necessary step for the proliferation of smooth muscle cells^88^. Thus, further research is needed to clarify the apparent discordant role on inhibition and proliferation of VASH1 on endothelial cells from hemangiomas. Of note, tubulin detyrosination is an important step during the Epithelial-to-Mesenchymal (EMT) transition in breast tumor cell metastasis^89^.

### Linearly responsive putative involution biomarkers

From the putative involution biomarker gene list, 50% have a linearly increasing response. More specifically, those genes are: ATPase ${{\rm{Na}}}^{+}$/${{\rm{K}}}^{+}$ transporting subunit $\beta$1 (*ATP1B1*), CD9 molecule (*CD9*), N-myc downstream regulated 1 (*NDRG1*), fibulin 1 (*FBLN1*), and secreted frizzled related protein 1 (*SFRP1*). The possible roles on IHs are described below.

#### ATP1B1

This gene encodes the $\beta$1 subunit of Na-K-ATPase (sodium pump) which is a cotransporter regulating electrolytes, cell volume and other signal transductions. Na-K-ATPase performs different roles in specific tissues. In the epithelia, Na-K-ATPase $\beta$1 subunit interactions are important for the stability of adherens junctions and tight junction integrity. Conditional knockout mice of Na-K-ATPase $\beta$1 in cardiomyocytes showed a reduction of cardiac contractibility and early collagen deposition^90^. Abnormal expression of Na-K-ATPase $\beta$ subunit has been reported in many carcinomas; in particular, decreased levels of Na-K-ATPase $\beta 1$ have been reported for both bladder carcinoma and clear cell carcinoma^91^. Thus, IHs may be mimicking some of cancer mechanisms (e.g., EMT) to proliferate or induce mobility via a reduction on expression of $\beta$1 subunit of Na-K-ATPases.

#### CD9

The tetraspanin molecule CD9 is expressed at a high level in endothelial cells. CD9 biological functions are based on its dynamic interaction with other trans-membrane or cytoplasmic proteins where it can promote or limit access to protein interactions^92^. Kamisasanuki and colleagues^93^ showed that CD9 plays a critical role in angiogenesis, and a CD9-knockdown mouse model had reduced proliferation of activated endothelial cells, whereas on quiescent endothelial cells its proliferative impact was lessened. In the same paper, it was observed that integrin $\beta 1$ was abnormally localized in an *in vitro* wound healing assay upon knocking down *CD9*. It is worth mentioning that the expression of *CD9* in our experiments is contrary to the angiogenic effect of CD9 presented by Kamisasanuki *et al*. One possible explanation is that the role of CD9 in hemangiomas is limited to the co-localization of integrin $\beta 1$ during the proliferative stage which induces angiogenesis, whereas in the involution phase CD9 angiogenic effect is diminished by the abundance of quiescent vasculature. However, more research is needed to clarify the role of CD9 on hemangiomas.

#### FBLN1

Fibulin 1 is an extracellular matrix glycoprotein associated with basement membranes which functions as an intramolecular bridge to stabilize ECM structures^94^. *In vitro* experiments conducted by Xie and colleagues^95^ demonstrated that Fibulin 1 inhibits endothelial cell proliferation and can inhibit tumor growth by suppressing angiogenesis and inducing apoptosis. Thus, it is possible that Fibulin 1 participates in the anti-angiogenic microenvironment during the involution phase of IHs.

#### NDRG1

This gene encodes the cytoplasmic protein NDRG1 that has been labeled as metastasis suppressor, and its elevated expression has been associated with improved survival in patients with grade II gliomas^96^. Further, *NDRG1* over-expressing malignant glioma cells exhibit reduced angiogenic activity^97^. NDRG1 is regulated by hypoxia, and by mTORC2 via SGK1 in glioma^96^. In a recent study, Byun *et al*.^98^ found that NDRG1 (and FOXO1) regulate endothelial cell proliferation in IHs; however, their results in hemangioma-derived stem cells show a different pattern of gene (and protein) expression than ours. If the gene expression follows Byun *et al*. findings, namely that *NDRG1* is up-regulated in the proliferating phase, then the general hypothesis that over-expression of NDRG1 is antiangiogenic and proliferation suppressor would be difficult to reconcile. Furthermore, supernatant of U87MG glioma NDRG1 knockdown contained proangiogenic proteins that increased sprouting in HUVEC cells^97^. This discrepancy could derive from the stemness of the endothelial cells studied under normal conditions (i.e., hyperoxia) that may disturb the network of interactions present in the hemangioma microenvironment. Nonetheless, it warrants further research to investigate the role of *NDRG1* on IHs.

#### SRFP1

The secreted frizzled related protein 1 (or FrzA) is a member of the group of secreted frizzled related proteins believed to work as an antagonist of Wnt signaling. It has been shown that this protein controls cell growth in an *in vitro* model of bovine aortic endothelial cells^99^. In mice, over-expression of SRFP1 was shown to induce a reduction of vascular cell proliferation after ischemic conditions^100^.

### Non-linearly responsive putative biomarkers

The analysis of biomarkers that follow non-linear responses represents a challenge, since the physiological environment (e.g., flow rates) changes over the course of the illness. Nonetheless, these subsets of biomarkers may hold important information about the development of IHs.

We will describe briefly only one putative biomarker that was present on both the proliferation and involution lists: periplakin.

#### PPL

Periplakin is a member of the plakins family that have numerous functions to link cytoskeletal elements together. Whereas most of the reports have concentrated on the role of periplakins in the epithelium, periplakins have been found to be expressed in endothelial cells. It has been suggested that periplakin act as a localization signal for protein kinase B (PKB), and thus it may be involved in many growth-factor induced cellular responses^101^. The role of periplakin on IHs remains an open question. Of note, the loss of periplakin has been associated with the pathological stage of urothelial carcinoma of the urinary bladder^102^. We confirmed that the relative expression of *PPL* was much higher for IHs in the involution phase (see Fig. 3).

## Conclusions

It has been more than a decade since the serendipitous finding by Léauté-Labrèze^11^ of propranolol for the treatment of infantile capillary hemangiomas. Despite the fact that the molecular mechanism of action of propranolol on endothelial cells has not been completely clarified, several high throughput experiments have been conducted to observe the mechanistic effects of propranolol exposure. The data integration used in this paper goes beyond classic meta-analysis as it places experimental results into a more coherent framework that requires not only a careful data selection but a systematic reanalysis (when raw data was available) from different sources and platforms that would ideally reduce experimental bias by cross referencing orthogonal methodologies. The result was a bioinformatically corroborated set of genes (which we refer to as *putative biomarkers*) that have consistently being reported as genes affected by propranolol treatment for infantile hemangiomas. Our data integration and analysis therein corroborated the involvement of hypoxia in IH development, and that hypoxia is driven by HIF-2α and not by HIF-1α. It is well stablished that HIF-2α levels are regulated by inflammatory, differentiation and stress signals, but it has never been involved in the development of IHs as we have convincingly demonstrated in our experiments. Moreover, in the malignant transformation of renal tubular cells changes in *HIF-2α* have been observed^103^, thus this research may have possible implications in other epithelial malignancies^17^. Another important finding was that propranolol-induces apoptosis (at least in the endothelium) via Myosin1b-Arg-II-mTORC1 or small calcium-binding mitochondrial carriers, thereby inducing mitochondrial stress. This finding contrast with other reports that propranolol induces apoptosis by G0/G1/S phase arrest in melanoma cells and liver cells^17,104^.

One major boon to the search for a putative miRNA signature would be to find a highly correlated expression in endothelial cells as well as on circulation. For instance, C19MC was proven to be secreted solely by GLUT-1^+^ IH endothelial cells, and was found also in the circulation^23^. Ideally, putative microRNA biomarkers for IHs would be able to trace the development of IHs through more accessible biological specimens (e.g., saliva) particularly in cases where responses to treatment are less apparent.

Propranolol treatment is now the first drug of choice for IHs^16,105^. However, lesion relapse after therapy remains an open question. While one can always argue that this is due to the individual’s genetic makeup (e.g. SNPs on key regulatory genes for propranolol biosynthesis), other possibilities may lie on the unique non-linear pattern of growth of IHs and concomitantly gene biomarkers. To convincingly answer these questions more research is warranted at the population level (e.g., GWAS) that can be further integrated and guide personalized treatment for children who experience side effects or are more susceptible to propranolol.

One limitation in our study is the reliance on genomics data and analysis. A full description of propranolol mechanism of action for IHs should ideally include proteomics, metabolomics or other modalities. Nonetheless, the gene lists presented here should provide a guide for future mechanistic studies on the effects of propranolol. However, such endeavors are beyond the scope of this article.

## Methods

### Subjects and tissue collection

This study was reviewed and approved by the Institutional Review Board of the University of Arkansas for Medical Sciences under the IRB number 228920. All methods performed on this research followed national regulations and guidelines for human subjects research protections. Under our collect and distribution protocol (IRB 114012), written informed consent from the parents and/or legal guardians of 28 children of various ages was obtained and then fresh tissue was harvested by surgical removal. Upon harvesting, tissues were stored at −80 °C until the RNA extraction was performed.

### IHs and propranolol samples

#### Samples preprocessing

Total RNA was isolated and purified from frozen tissues using the RNeasy Plus Mini Kit (Qiagen,Valencia, CA). The Illumina TotalPrep RNA amplification kit (Illumina, San Diego, CA) was used to prepare biotinylated antisense cRNA from 500 ng of high-quality total RNA for subsequent global gene expression profiling by the Pharmacogenomics Analysis Laboratory (Central Arkansas Veterans Healthcare System, Little Rock, AR). Quality and quantity of cRNA were determined by Ribogreen fluorescence and Agilent bioanalyzer electropherograms (Agilent Technologies, Santa Clara, CA). A total of 750 ng of cRNA per sample was loaded onto the Illumina Human HT-12 v4 Gene Expression BeadChip for hybridization at 58 °C for 17 hours. After blocking, staining, and washing, the microarrays were scanned on the Illumina iScan system. Data on gene-level intensities was extracted using BeadStudio (Illumina). The detection $$p$$-values and average number of beads per gene were extracted with the *BeadArray* package from Bioconductor. Since the background was subtracted with BeadStudio, the negative controls were estimated from the data itself. The neqc function in the *limma* package was used to apply a normal-exponential convolution model for background correction followed by quantile normalization and logarithmic transformation to the intensities. A non-specific filter was applied on genes whose detection was statistically significant ($$p$$-value $$ < 0.05$$) in at least three samples.

#### Differential gene expression

We used the Bioconductor *limma* package to determine differential gene expression. We fit a linear model that encodes the difference between two consecutive experimental time points, i.e., contrasts between time $t$ versus time $t-1$. Then we applied an empirical Bayes method to moderate standard errors of the estimated $\log$-fold changes. Probes were selected based on an FDR-adjusted $p$-value of $0.05$.

Other routines and packages from Bioconductor were used for the analysis of microarray chips, and they were briefly outlined on the results section.

#### Gene expression validation by real-time quantitative PCR

To validate our results, we conducted RT-PCR on a second set of biopsies from patients clinically diagnosed with IHs in the proliferative phase (less than 6 mo) or in the involution phase (more than 24 mo). Total RNA was isolated using the RNeasy Plus Mini kit (Qiagen, Valencia, CA) according to the manufacturer’s instructions. Extractions were treated with RNase-free DNAse to eliminate unwanted DNA. Concentrations (ng/µL) and O.D. ratios (260/280 nm) of total RNA were determined using the Nanodrop UV/VIS spectrophotometer (Thermo Fisher Scientific, Waltham, Massachusetts). RNA Integrity Numbers (RIN scores), which are a ratio of ribosomal RNAs 18S and 28S in human samples, were obtained using the Agilent 2100 Bioanalyzer with the Agilent RNA 6000 Nano Kit (Santa Clara, CA). All total RNA specimens had O.D. ratios between 1.85-1.99 with RIN scores of >8.0. The quantitative conversion of 400ng of total RNA to single-stranded cDNA in a single 40 µL reaction was performed with the High Capacity cDNA Reverse Transcription Kit (Applied Biosystems, Foster City, CA). Quantitative (q)PCR was performed using the Applied Biosystems 7900HT real-time PCR system with SDS v2.4.1 software. All qPCR reactions were carried out in a final volume of 10 μl containing 1x of TaqMan™ Gene Expression Master mix with UNG (Applied Biosystems), 1x of each TaqMan™ Gene Expression Assay (FAM-MGB dyes), and 20 ng cDNA in sterile molecular-grade water (see Table 6). The standard cycling condition was 50 °C for 2 min, 95 °C for 10 min, followed by 40 cycles of 95 °C for 15 s and 60 °C for 1 min. qPCR was performed in triplicate to ensure quantitative accuracy and the Ct threshold was 0.09. The results were analyzed using SDS V12.2 relative quantification manager software. The comparative threshold cycles values were normalized for Beta-Actin reference genes. Relative changes were calculated using the ${2}^{-\varDelta \varDelta Ct}$ method.

**Table 6:** TaqMan Gene Expression Assays.

Assay ID	Gene Symbol	Availability
Hs01026149_m1	EPAS1	Inventoried
Hs00196221_m1	LASP1	Inventoried
Hs01012756_m1	SLC25A23	Inventoried
Hs00362654_m1	MYO1B	Inventoried
Hs00946916_m1	ALDH1A1	Inventoried
Hs01011417_m1	PPL	Inventoried
Hs99999903_m1	ACTB	Made to Order

## Data Availability

The data generated in this publication has been deposited in NCBI’s Gene Expression Omnibus and is accessible through GEO Series accession number *GSE127487*.
